# Reactividad del cortisol y salud mental en adultos expuestos a violencia temprana: revisión sistemática

**DOI:** 10.26633/RPSP.2017.171

**Published:** 2017-12-26

**Authors:** Ana Lilia Cerda-Molina, Javier Iván Borráz-León, Lilian Mayagoitia-Novales, Alina Teresita Gaspar Del Río

**Affiliations:** 1 Instituto Nacional de Psiquiatría Ramón de la Fuente Muñiz Instituto Nacional de Psiquiatría Ramón de la Fuente Muñiz Ciudad de México México Instituto Nacional de Psiquiatría Ramón de la Fuente Muñiz, Ciudad de México, México.

**Keywords:** Hidrocortisona, salud mental, violencia familiar, violencia sexual, abuso sexual infantil, trastornos por estrés postraumático, sistema hipófiso-suprarrenal, depresión, Hydrocortisone, mental health, domestic violence, sex offenses, child abuse, sexual, stress disorders, post-traumatic, pituitary-adrenal system, depression, Hidrocortisona, saúde mental, violência doméstica, delitos sexuais, abuso sexual na infancia, transtornos de estresse pós-traumáticos, sistema hipófise-suprarrenal, depressão

## Abstract

**Objetivo.:**

*Analizar los resultados con respecto a la reactividad del cortisol (RC) ante un paradigma de estrés experimental en adultos con o sin algún trastorno psiquiátrico que sufrieron violencia en etapas tempranas de desarrollo (infancia y adolescencia temprana) y con ello proponer una herramienta clínica para el establecimiento de indicadores biológicos de estrés*.

**Métodos.:**

*Se realizó una revisión sistemática en diversas bases de datos, siguiendo los criterios PRISMA; de un total de 231 artículos, 16 cumplieron los criterios de inclusión y los resultados fueron analizados de manera cualitativa*.

**Resultados.:**

*A pesar de la heterogeneidad, los resultados sugieren que las personas que sufrieron violencia temprana presentan un patrón de baja RC. Contrariamente, la población que además desarrolló síntomas de trastorno de estrés postraumático y depresión, independientemente del tipo de violencia, mostró incrementada RC. La mayoría de los trabajos se centraron en población que sufrió abuso sexual en la infancia y la mitad de los artículos apoya la hipótesis de que la RC es más pronunciada en hombres que en mujeres*.

**Conclusiones.:**

*Los resultados de esta revisión nos permiten sugerir que es posible considerar la hiperreactividad del cortisol como un biomarcador para el tratamiento e intervención de población con trastorno de estrés postraumático y depresión que sufrieron violencia temprana. Además, apoyan la evidencia de que sufrir violencia altera la respuesta del estrés y la salud mental a largo plazo. Sin embargo, es necesario realizar más estudios principalmente los que se refieren a la hiporreactividad y a las diferencias de género*.

## Abstract (en)

**Objective.:**

Analyze results with respect to cortisol reactivity (CR) in experimental stress paradigms in adults with or without a psychiatric disorder who suffered violence in early developmental stages (infancy or early adolescence); and use these results to propose a clinical tool to determine biological stress indicators.

**Methods.:**

A systematic review was conducted using several databases and following PRISMA criteria; of a total of 231 articles, 16 satisfied the inclusion criteria and the results were analyzed qualitatively.

**Results.:**

Despite heterogeneity, the results suggested that the people who suffered violence at an early age present a pattern of low CR. In contrast, people who also developed symptoms of post-traumatic stress disorder and depression, regardless of the type of violence, showed increased CR. The majority of studies focused on people who suffered childhood sexual abuse and half of the articles supported the hypothesis that CR is more pronounced in men than in women.

**Conclusions.:**

The results of this review suggest that cortisol hyperreactivity can be considered a biomarker for treatment and intervention in people with post-traumatic stress disorder and depression who suffered violence at an early age. The results also support evidence that experiencing violence alters stress response and mental health in the long term. However, it is necessary to conduct more studies, in particular studies on hyporeactivity and gender differences.

## RESUMO

**Objetivo.:**

Analisar os resultados da reatividade do cortisol segundo um modelo de estresse experimental em adultos com ou sem transtorno psiquiátrico que foram precocemente expostos à violência durante o desenvolvimento (infância e início da adolescência) e propor um instrumento clínico para determinar indicadores biológicos de estresse.

**Métodos.:**

Uma revisão sistemática foi conduzida em diferentes bases de dados conforme os critérios PRISMA. Dos 231 artigos selecionados, 16 satisfizeram os critérios de inclusão. Foi realizada uma análise qualitativa dos resultados.

**Resultados.:**

Apesar de serem heterogêneos, os resultados indicam que adultos com exposição precoce à violência apresentam baixa reatividade do cortisol. De maneira oposta, os indivíduos que desenvolveram sintomas de transtorno de estresse pós-traumático e depressão, independentemente do tipo de violência sofrida, apresentaram elevada reatividade do cortisol. A maioria dos estudos se centrou em vítimas de abuso sexual na infância e, em metade dos artigos, sustentou-se a hipótese de que a reatividade do cortisol é mais acentuada no sexo masculino que no feminino.

**Conclusões.:**

Os resultados deste estudo de revisão apontam que se pode considerar a hiper-reatividade do cortisol como biomarcador para o tratamento e a intervenção de pacientes que foram expostos precocemente à violência e que apresentam transtorno de estresse pós-traumático e depressão. Além disso, é reforçada a evidência de que sofrer violência afeta a resposta ao estresse e a saúde mental a longo prazo. Porém, fazem-se necessários mais estudos, sobretudo investigando hiporreatividade e diferenças de gênero.

La violencia es un problema de salud pública, responsable de variadas consecuencias negativas y alteraciones en la salud mental y física de la población (1,2). Diversos estudios epidemiológicos indican que la exposición a la violencia en la infancia y/o adolescencia constituye un factor de riesgo para experimentar trastornos del estado de ánimo y de ansiedad, estos se pueden expresar en el corto o largo plazo e incluso perdurar toda la vida (2-5). Mundialmente se calcula que una cuarta parte de todos los adultos han sufrido de maltratos físicos durante la infancia, de estos, 1 de cada 5 mujeres y 1 de cada 13 hombres declaran haber sufrido abusos sexuales (1). El maltrato infantil conduce a importantes afectaciones en la salud física y mental de la población, particularmente en ambientes de pobreza (6-8).

Experimentar situaciones de violencia y trauma activan respuestas fisiológicas de estrés agudo, que de vivirse de manera repetida, se vuelve crónico (9,10); existen evidencias de que el estrés en etapas tempranas de la vida, predispone a las personas a sufrir patologías afectivas que se ven reflejadas en la disfunción de la respuesta neuroendocrina al estrés mediada por el eje hipotálamo-hipófisis-adrenal (HHA) y el sistema nervioso simpático (11). En respuesta a un estresor, se secreta la hormona liberadora de corticotropina (CRH), la cual estimula en la adenohipófisis la secreción de la hormona adrenocorticotrópica (ACTH), que a su vez estimula la secreción de cortisol en la corteza suprarrenal (12,13). El cortisol, es una hormona esteroide que ejerce funciones adaptativas para responder a estímulos adversos tales como: movilización de glucosa, incremento en la actividad muscular y del tono cardiovascular, secreción de ácidos gástricos, etc. (14,15). Una vez que la situación de peligro ha pasado, el propio cortisol ejerce un mecanismo de retroalimentación negativa para volver al estado basal y restablecer la homeostasis del cuerpo; no obstante, la repetición del evento genera una sensibilización del sistema de regulación de la CRH, y por lo tanto, el sistema de retroalimentación se altera (16). La sensibilización persistente de este sistema, constituye un substrato biológico para desarrollar una mayor vulnerabilidad a sufrir estrés subsecuente, daños en el sistema cognitivo-emocional y en consecuencia, desarrollar algún trastorno mental a largo plazo (10, 17, 18). Muchos estudios experimentales clínicos y no clínicos, sugieren que la violencia temprana y el estrés inducen una respuesta alterada de la secreción de la CRH y de otros sistemas de neurotransmisión involucrados en la recompensa y la tendencia a las adicciones (19, 20). No obstante, el sentido de la alteración de la reactividad del cortisol (RC) en los casos de violencia temprana aún no está bien esclarecido ya que hay literatura que afirma que la activación persistente del eje HHA durante la infancia conduce al desarrollo, durante la etapa adulta, de trastorno de estrés postraumático (TEPT), episodios depresivos y de ansiedad (21-23), y hay literatura que muestra que la alteración de la RC es independiente del desarrollo de algún trastorno (24). Por ejemplo, Heim y cols. (25) describieron que mujeres adultas que padecían depresión (DD) y que habían sido víctimas de abuso sexual en la infancia, mostraron hiperreactividad del eje HHA y, por lo tanto, mayor secreción de cortisol ante una prueba de estrés, mientras que las mujeres que sufrieron abuso pero que no presentaron síntomas depresivos, tuvieron un patrón de secreción de cortisol similar al de mujeres saludables, es decir, un pico de cortisol moderado. Contrariamente, Juul y cols. (24) describieron que mujeres adultas con reporte de abuso sexual y otros traumas durante la infancia, disminuyeron su RC y aunque algunas mujeres presentaban DD, este trastorno no fue predictivo de la respuesta. También, se han descrito diferencias de acuerdo al tipo de prueba de inducción de estrés de laboratorio utilizado (si es de tipo cognitivo o emocional) y del sexo (16, 26-29).

El objetivo de esta revisión sistemática fue analizar los resultados con respecto a la RC ante un paradigma de estrés experimental en adultos con o sin algún trastorno psiquiátrico, qué sufrieron estrés crónico generado por escenarios de violencia en etapas tempranas de desarrollo (antes de los 16 años), para aportar datos que nos permitan esclarecer la discusión de los resultados contradictorios y sugerir la posible utilidad de la RC en la clínica como biomarcador para establecer el tipo de intervención e incrementar la efectividad del tratamiento.

## MATERIALES Y MÉTODOS

La estrategia de búsqueda bibliográfica se realizó siguiendo los criterios PRISMA de artículos, con el tema de reactividad del cortisol en adultos (18 años en adelante) que sufrieron violencia durante la infancia y adolescencia temprana. Los términos de búsqueda en inglés fueron: *violence exposure* and *cortisol reactivity* con la siguiente ruta de búsqueda: en MEDLINE (“violence”[MeSH Terms] OR “violence”[All Fields]) AND exposure [All Fields] AND (“hydrocortisone”[MeSH Terms] OR “hydrocortisone”[All Fields] OR “cortisol”[All Fields]) AND reactivity [All Fields]. También se utilizó el término *cortisol response* y *adrenal reactivity* para ampliar las opciones de búsqueda del término reactividad. Se utilizó la palabra violencia como MeSH dado que el término involucra todos los tipos de trauma, maltrato y abuso. Los equivalentes de los mismos términos se usaron para la búsqueda en español. Se consultaron las siguientes bases de datos: PubMed, CONRICYT, Web of Science, PsycINFO, SciELO y LILIACS. La búsqueda se realizó en enero de 2016; se limitó a trabajos publicados a partir del año 2000, en seres humanos y a revistas académicas de revisión por pares.

Los criterios de inclusión fueron: estudios transversales o longitudinales, con población clínica y no clínica, población adulta (≥ 18 años), hombres y mujeres con reporte de algún tipo de violencia o trauma temprano (antes de los 16 años) sin importar la duración de la exposición que hubiesen medido la RC (en saliva o suero) ante una prueba de estrés de laboratorio (utilizando una o más muestras basales y una o más muestras post-estresor), y con reporte o no de algún desorden psiquiátrico medido por medio de inventarios estructurados (*SCID-DSM-IV, Inventario de Depresión de Beck, Escala Hospitalaria de Ansiedad y Depresión (HADS), la Escala para Depresión de Hamilton y Escala clínica de TEPT (CAPS)*)**. Debido a que no hay un consenso en el rango de edad para el término *violencia temprana*, se estableció arbitrariamente los 16 años, que corresponde al final del periodo de adolescencia media y suponiendo que dos años es suficiente para desarrollar un posible trastorno psiquiátrico, antes de la edad mínima de inclusión para la realización de la prueba de RC.

Para garantizar la calidad de los artículos se consideraron sólo aquellos que reportaron los datos de la población de estudio, el lugar, la fuente de financiamiento, la aprobación de los Comités de Ética Institucionales, de los reactivos utilizados y los coeficientes de variación, así como aquellos que controlaron de manera previa variables de confusión que afectan la medición de cortisol, como no haber comido, tomado café o fumado, al menos dos horas antes de la prueba y no haber tomado medicamentos el día del procedimiento. No se restringió la hora en la que se hicieron las mediciones, dado que trabajar con población clínica puede dificultar la organización de los procedimientos experimentales.

Se excluyeron trabajos que únicamente consideraron el análisis del ritmo diurno de cortisol, de RC medida en adolescentes o niños, de RC en pacientes con TEPT o DD cuando no hubo reporte de violencia o trauma en etapas tempranas o si los desórdenes psiquiátricos mencionados fueron consecuencia de algún desastre ambiental o evento reciente.

**FIGURA 1.**
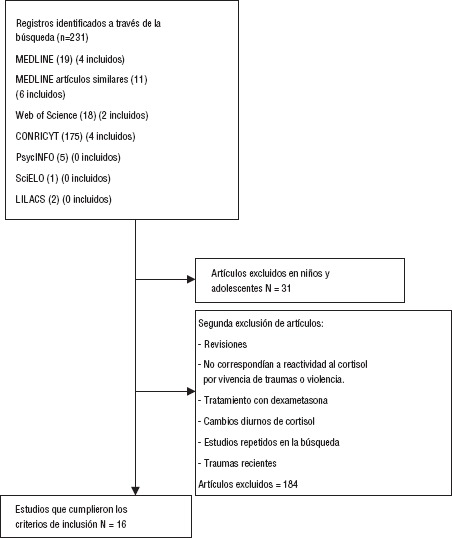
Diagrama PRISMA con la historia de búsqueda, hasta abril de 2017, para reactividad del cortisol

Para la colección de los datos y el análisis, se construyó un diagrama PRISMA (figura 1) en el que se anotó el número de artículos encontrados por búsqueda y cuáles ingresaron al estudio a partir de los criterios de inclusión y exclusión.

Los autores Cerda-Molina A.L. y Borraz-León J., revisaron los resúmenes y la metodología de los artículos para decidir su inclusión. Un tercer autor, Mayagoitia-Novales L., realizó una comprobación de la inclusión de los artículos seleccionados y se obtuvo un índice Kappa de 0,89. Todos los artículos se obtuvieron en texto completo por los siguientes medios: acceso gratuito, *research gate*, claves de acceso institucional y solicitud vía correo electrónico a los autores. Se elaboró un cuadro con el resumen de los resultados para su análisis cualitativo (cuadro 1). No se realizó un metanálisis porque sólo 5 artículos incluían datos de media de cortisol y desviación estándar o intervalos de confianza (cuadro 2); de estos, sólo 2 mostraron el valor original, y el resto reportó medias residuales o de valores transformados a logaritmo. Otros autores mostraron datos de media general, sin distinguir entre la población que sufrió o no violencia, la población control y/o la población con algún trastorno. Por tal motivo se analizaron de manera cualitativa los resultados principales de la RC y qué variables podrían explicar los resultados contradictorios.

## RESULTADOS

Se identificaron un total de 16 artículos que cumplieron con los criterios de inclusión, todos ellos en idioma inglés. Los estudios incluyeron en total 1 707 personas y/o pacientes, de los cuales 1 246 fueron personas que reportaron haber experimentado violencia y el resto fueron controles. Los tipos de violencia que se describieron fueron: abuso sexual, adversidades acumuladas (i.e., abuso sexual + abuso emocional y físico + maltrato infantil + amenazas, etc.), experiencias de tortura y guerra en poblaciones de refugiados y en sobrevivientes del Holocausto, vivir en ambientes de riesgo con pobreza y negligencia, e historia de agresión familiar.

De los 16 artículos (cuadro 1), en 12, se estudiaron personas expuestas a abuso sexual, que en algunos casos reportaron otras adversidades (7, 21, 24, 25, 30-37) De estos 12 artículos, 9 incluyeron personas que presentaban TEPT o DD (21, 24-25, 30, 32-35, 37). Los estresores de laboratorio reportados fueron: pruebas cognitivas (TSST, por sus siglas en inglés *Trier Social Stress Test* o variantes del mismo), entrevistas estructuradas, conflictos interpersonales provocados, y el paradigma de separación-reunión del bebé, en binomios madre-infante.

Todos los artículos incluidos en la revisión, con excepción del de van der Hal-Van Raalte et al. (38), contaron con información sobre controles de calidad y coeficientes intra-e inter-ensayo del reactivo hormonal utilizado y la hora del día.

No se contó en todos los casos con población saludable o no expuesta a violencia, como grupo control o de comparación. De los 16 artículos, 9 no consideraron población control alguna (7, 21, 30, 32-33, 37-40) y 7 sí lo hicieron, pero en 2 de ellos (31, 41) el control fue otro grupo con exposición a violencia y sin la aplicación de la prueba de estrés, 2 con población sin violencia pero con algún trastorno (24, 37) y sólo en 3 estudios la población de comparación fue saludable (25, 34, 37).

Con respecto al patrón de secreción de cortisol, encontramos que en 6 de 12 estudios que incluyeron a personas con historia de abuso sexual, más otras adversidades acumuladas y TEPT o DD, la RC fue elevada, en comparación con personas saludables, o en comparación con personas violentadas pero sin los trastornos (21, 25, 30, 34-35, 37). La excepción a este patrón de hiperreactividad fueron 2 trabajos donde a pesar del abuso sexual y de haber desarrollado TEPT y DD, la respuesta fue de atenuación y disminución de la RC (24, 33). En las personas con historia de abuso sexual y maltrato que no padecían ningún trastorno la respuesta también fue atenuada o disminuida (30-32, 35). Otro estudio donde se observó hiperreactividad, fue en la población de sobrevivientes del Holocausto (38); en este estudio los hombres con TEPT presentaron mayor RC, que los que no padecían el trastorno.

**CUADRO 1.** Resumen de los resultados descritos en los artículos de la reactividad del cortisol ante un estresor de laboratorio en adultos expuestos a violencia temprana

Autor	Tipo de violencia/trastorno	N (población expuesta)	Edad (años), media ± SD y/o rango y/o CI	N (control)	Edad (rango y/o media ± SD	N total (H/M)	Resultado	Diferencias por sexo
Aiyer *et al*. (7)	Adversidades acumuladas (abuso físico, emocional y sexual, falta de apoyo familiar)	266	14,9 ± 0,64 (al inicio del estudio). 22,1 ± 0,66 (prueba de estrés).			43%/57%	Sólo a niveles bajos de apoyo del padre, la violencia acumulada se asoció con una **disminución** pronunciada de la RC	Menor en hombres
Elzinga *et al*. (21)	Abuso sexual y físico/TEPT, DD	24 (12 con TEPT, 12 sin TEPT)	TEPT = 35,67 ± 9,94, sin TEPT 38,75 ± 10,47 (SEM)			0/24	Las mujeres con TEPT presentaron **aumento** en la RC en comparación con las mujeres sin TEPT.	NA
Juul *et al*. (24)	Traumas infantiles y abuso sexual/DD	40 Binomio Madre-infante 85.7% DD	33,36 ± 4,43	152 Diadas Madre-infante 81.1% DD	33,81 ± 4,37	0/192	**Disminución** de la RC que a su vez, predijo mayor afecto neutro durante la interacción madre-infante. La DD o distimia no fueron predictores de la RC	NA
Heim *et al*. (25)	Abuso sexual/DD	27 (13 con DD, 14 sin DD)	DD = 32 IC 27-36, sin DD = 30 IC 27-33	12 SS, 10 DD	SS = 29 IC 24-34, DD = 34,6 IC 28,59-40,60	0/49	Mujeres con DD mostraron **elevada** RC en comparación con todos los grupos	NA
Schechter *et al*. (30)	Abuso sexual y físico, violencia doméstica/TEPT	36 Binomio Madre-infante con TEPT	30 19-45 (Rango)			0/36	Madres con TEPT severo tuvieron **elevada** RC; las madres sin TEPT disminuyeron la RC;	NA
Lovallo *et al*. (31)	Adversidades (abuso sexual, asalto, amenaza con armas, separación materna).	354 ^*^ 0 = 164 1 Adversidad = 122 >1 Adversidades = 68	Hombres 0 = 23,5 ± 0,3 1= 23,7 ± 0,6 >1= 24,6 ± 0,7 Mujeres 0 = 23,1 ± 0,3 1= 23,5 ± 0,4 >1= 24,6 ± 0.5	La misma población sin el test de estrés		158/196	A mayor número de eventos adversos, la respuesta de la RC fue **disminuida** (0>1>2 o más eventos) para ambos sexos	Mayor en hombres
Elzinga *et al*. (32)	Adversidades acumuladas (abuso físico, emocional y sexual)/DD, AS	77 (32 Alto nivel 45 Bajo nivel)	21,6 ± 3,61 Alto = 21,79 ± 3,71, Bajo = 21,47 ± 3,58			49/28	Ambos grupos presentaron RC pero la respuesta fue **atenuada** en el grupo con altos niveles de adversidad.	Mayor en hombres
Schalinski *et al*. (33)	Adversidades (abuso físico, emocional y sexual)/TEPT, DD	43 21 con abuso sexual	34,9 ± 10,5			0/43	Personas con abuso sexual presentaron **disminución** de la RC en comparación con las personas sin abuso. El efecto se conservó excluyendo a los pacientes sin TEPT	NA
Bremner *et al*. (34)	Abuso sexual/TEPT	23 TEPT	43 ± 10	18 SS	49 ± 10	16/25	**Elevada** RC en personas con TEPT	Mayor en hombres
Brand *et al*. (35)	Abuso sexual/TEPT, DD	126 Binomio Madre-infante: (28 abuso sexual; 15 TEPT, 55 estrés pre-postnatal, 38 DD )	34 ± 4,0	70% de las mujeres sin abuso.		0/126	Las madres con abuso sexual presentaron d**isminución** en la RC. Las madres con abuso sexual con DD, estrés o TEPT presentaron **aumento** en la RC	NA
Carpenter *et al*. (36)	Maltrato (abuso físico, sexual y emocional, negligencia)	23 (sólo 9 con abuso sexual)	35,0 ± 12,9	27 SS	24,1 ± 6,6	17/33	Respuesta **atenuada** de la RC comparado con el grupo que no sufrió maltrato.	NO
Gola *et al*. (37)	Refugiados con historia de tortura y violación/TEPT, DD	30 TEPT 10 con violación	Con violación = 34 ±10,2; sin violación = 33,7 ± 8,7			16/14	Los refugiados con TEPT y violación mostraron **incremento** en la RC. El grupo sin violación presentó **disminución** en la RC, en ambos sexos	NO
van der Hal-Van Raalte *et al*. (38)	Sobrevivientes del Holocausto nacidos entre 1935 y 1944/TEPT, DD	133	64,6 ± 2,94 61-68			39%/61%	Los sobrevivientes más jóvenes tuvieron la RC más **elevada**. Los hombres con TEPT tuvieron **mayor** RC que los hombres sin TEPT	Mayor en hombres
Brenner *et al*. (39)	Vivir en zonas de riesgo por pobreza	163	21 ± 0,63			50%/50%	Niveles elevados de pobreza y estrés **disminuyeron** la RC. Niveles bajos de pobreza y estrés aumentaron la RC	NO
Aloia and Solomon (40)	Agresión Familiar	100 (50 parejas)	20,93 ± 0,99 18-31			50/50	Niveles altos de agresión familiar resulto en **disminución** de la RC, y contrario, bajos niveles de agresión, resulto en elevada RC.	NO
Kolassa *et al*. (41)	Refugiados con experiencia de guerra y tortura/TEPT	33 con TEPT	34 ± 7,6 22-50	De los 33 pacientes, 16 sin el test de estrés		33/0	La RC **disminuyó** en ambos grupos con y sin la entrevista sobre experiencias traumáticas	NA

AS = Ansiedad; C = Cortisol; DD = Depresión; IC = Intervalo de Confianza; NA = No aplica; RC = Reactividad del cortisol; TEPT = Trastorno de estrés post-traumático; SS = Saludable.

* El dato está dividido por número de situaciones adversas experimentadas. La respuesta disminuida hace referencia a hipo-reactividad, la respuesta aumentada o de incremento, a hiper-reactividad, la respuesta de atenuación, se refiere a un pico moderado de cortisol.

***Fuente:*** elaboración propia

**CUADRO 2.** Artículos que proporcionan los valores de la media de cortisol por grupo de estudio

Autor	Unidades Cortisol	Media (M)	Comparación	Control	Violencia (+DD o PTSD)
Heim *et al*. (25)	nmol/l	M (IC 95%)	Pico de C	Saludable 339 (281-395)	Con DD ^a^ 527 (455-596)
Schechter *et al*. (30)	µg/dl	M	Basal vs. Pico de C	Con TEPT ^a^ 1,97 vs. 1,14	
Schalinski *et al*. (33)	nmol/l	M (SD) valor en Log	C basal 40 min Fin test 1 Fin test 2	Sin abuso sexual 2,06 (0,4) 2,10 (0,61) 2,12 (0,6) 1,99 (0,53)	Con abuso sexual ^b^ 2,08 (0,38) 1,65 (0,32) 1,63 (0,34) 1,78 (0,51)
					Con TEPT ^a^ -0,56 (1,07) -0,65 (1,16) -0,69 (1,17)
Carpenter *et al*. (36)	µg/dl	M (SD)	Basal vs. Pico de C	412,1 (38,9) vs. 583,8 (37,4)	329,5 (42,6) vs. 412,8 (40,9) ^b^
van der Hal-Van Raalte *et al*. (38)	NA	M residual (SD)	C cada 20 min.	Sin TEPT -0,60 (1,06) -0,57 (1,02) -0,58 (1,00)	

C = Cortisol; IC = Intervalo de Confianza; Log = Logaritmo; SD = Desviación estándar.

a = Tendencia del cortisol a aumentar; b = Tendencia del cortisol a disminuir.

b = Tendencia del cortisol a disminuir.

***Fuente:*** elaboración propia

En los 4 estudios donde el tipo de violencia fue vivir en zonas de pobreza con poco apoyo paterno y negligencia (7, 39), con vivencia de guerra y tortura (41) y con historia de agresión familiar (40), la RC disminuyó o se atenuó.

Al analizar el efecto del tipo de prueba de estrés de laboratorio utilizada, se observó que los patrones antes citados de RC fueron independientes del tipo de estresor. El abuso sexual y el desarrollo de TEPT o DD provocaron hiperreactividad del cortisol, independientemente del estresor. En los 3 artículos donde citan mujeres con historia de abuso sexual y aplicación de pruebas de separación-reunión del bebé, se obtuvo un resultado consistente: RC elevada donde hubo síntomas de TEPT, y baja cuando no hubo dicho trastorno.

Un total de 9 artículos incluyeron ambos sexos en sus procedimientos, aun en los reportes de abuso sexual. De estos artículos, 4 no reportan diferencias por género (36-37, 39, 40) y los restantes 5 reportan que el efecto del cortisol fue más pronunciado en los hombres o incluso sólo significativo en ellos (7, 31-32, 34, 38).

Al analizar otras variables que pudieran sesgar los resultados como la hora del día en que se realizó el procedimiento, se observó heterogeneidad. Tres trabajos comenzaron a las 10:00, 2 no especifican la hora, 2 utilizan un rango de 9:00-14:00 y el resto se realizó entre las 13:00 y 18:00. De acuerdo al estudio de Kirchbaum y cols. (42), después del pico matutino de cortisol que ocurre alrededor de las 8:00, la hormona comienza a disminuir de manera gradual hasta valores relativamente estables alrededor de las 11:00. Sin embargo, aun los estudios que reportaron iniciar a las 9:00 (30-31), describieron resultados congruentes con los demás artículos. En el estudio de Elzinga y cols. (32), la RC se determinó a diferentes horas, sin reportar alteraciones en el patrón de respuesta

Con respecto al tamaño de la muestra encontramos heterogeneidad, con un mínimo de tamaño de muestra de 24 personas y un máximo de 354, con una media de 107,31 ± 95,5 (SD); sólo 2 artículos citaron valores *d* de Cohen (25, 33). Sólo un artículo fue de tipo longitudinal (7) e incluyó la edad de registro de la violencia (14 años).

## DISCUSIÓN

Esta revisión apoya la evidencia de que la violencia y el trauma experimentados en etapas tempranas de la vida alteran la salud mental y el eje HHA a largo plazo, por lo tanto, el patrón de secreción del cortisol puede considerarse como un biomarcador para algunos desórdenes psiquiátricos relacionados con el estrés. Podemos sugerir que la hiperreactividad del cortisol puede considerarse como un marcador de estrés en la mayoría de los casos de mujeres que desarrollaron DD y en los casos de personas con TEPT. Una característica de las personas que presentan algún desorden mental es que se observa un deterioro progresivo en la organización de sus sistemas biológicos y psicológicos, ante el esfuerzo de adaptarse adecuadamente a experiencias estresantes y adversas (43).

Más de la mitad de los artículos encontrados se centraron en el estudio del abuso sexual, lo que resalta la vulnerabilidad de la población infantil y la necesidad de incrementar las medidas de prevención, así como de detección y tratamiento tempranos. Los resultados observados en esta revisión, mostraron que en la mayoría de los casos el patrón de secreción de cortisol no dependió del tipo de violencia o del tipo de prueba de estrés; en cambio, la presencia de síntomas de TEPT y DD, en la mayoría de los casos, fue característica de una respuesta elevada del cortisol e hiperreactividad. Este resultado apoya la hipótesis de la desregulación de la retroalimentación negativa del cortisol, generando una respuesta incrementada de secreción de CRH, ante un estresor de naturaleza incontrolable, que amenaza la integridad, como lo es el abuso sexual (26, 29, 34).

Dos de los trabajos con resultados consistentes y que apoyan los hallazgos anteriores fueron realizados en población de madres con historia de abuso sexual infantil a las que se les aplicó la prueba de estrés de separación-reunión con el infante (30, 35). Ambos estudios describieron alta RC cuando hubo síntomas de TEPT y DD, pero baja RC cuando no hubo dichos síntomas. Por el contrario, en personas con antecedentes de vivir en zonas de pobreza y con negligencia, abuso emocional, con historia de agresión en la familia y con adversidades acumuladas incluido el abuso sexual, pero sin desarrollar TEPT, o DD, la tendencia fue a presentar hiporreactividad (7, 31, 36, 39-40). En condiciones normales, la secreción de cortisol es esencial en la capacidad individual para contender con eventos estresantes. El mecanismo mediante el cual una persona que sufrió algún trauma repetido durante la infancia y que en la etapa adulta muestra una RC baja sin desarrollar algún desorden psiquiátrico, no se conoce todavía, pero puede tratarse de una respuesta de habituación o protección a sufrir estrés crónico, con un efecto de regulación negativa de la respuesta de secreción de la CRH, así como a factores genéticos que aportan al individuo una mayor capacidad de resiliencia (7, 44-45). Por otra parte, en los dos estudios con personas refugiadas con antecedentes de violencia, encontramos algunos datos contrarios. Kolassa y cols. (41) describieron disminución de la RC en personas con TEPT (refugiados), no obstante en este estudio no se reportó que la población sufriera de abuso sexual como ocurrió en el de Gola y cols. (37) donde sí se reporta elevada RC (también en refugiados). Otro trabajo con resultados contradictorios fue el de Schalinski y cols. (33), con población expuesta a adversidades, entre ellas abuso sexual y con síntomas de TEPT y DD, en el que encuentran RC disminuida. Una de las variables que podría explicar los resultados contradictorios, es la variabilidad genética de la población, que confiere mayor capacidad de resiliencia, la edad en la que ocurrieron los abusos o el tiempo de exposición a la violencia. Por lo anterior, se plantea la necesidad de realizar más estudios que muestren si esta habituación que genera hiporreactividad, lejos de ser dañina, podría representar un factor de protección. Algunos estudios sugieren que los niños que están expuestos a violencia temprana, como víctimas o como testigos, se habitúan, y en consecuencia, dejan de ver a la violencia como un evento adverso (46); por ello, la respuesta emocional y endocrina al estrés cuando son adultos, es de atenuación.

Los desórdenes asociados al estrés, como el TEPT, se caracterizan por presentar bajo umbral al estrés, alto nivel de excitabilidad, tensión, fatiga y dolor (47-48); lo cual explica, la hiperreactividad del eje HHA observado en esta revisión. Esta alteración tiene consecuencias serias para la salud, pues en algunos trabajos se ha asociado con susceptibilidad a padecer enfermedades infecciosas y problemas de tipo cardiovascular (11).

Cabe resaltar que en las búsquedas no se encontraron artículos realizados en Latinoamérica (aunque algunos estudios contemplaron población hispana), donde las situaciones de violencia son cada vez más pronunciadas, por lo que es necesario llevar a cabo investigaciones sobre este tema en la región. Con respecto a las diferencias por sexo, tal como se ha mostrado en la literatura, básicamente del grupo de Kirchbaum (27-28, 42), los hombres presentaron un incremento de la RC comparado con el de las mujeres, aunque no se reportó tal diferencia en casi la mitad de los artículos que incluyeron el sexo como variable. Esta diferencia sexual se ha asociado a la secreción de estrógenos en las mujeres (49). Cabe señalar que hacen falta más estudios que aclaren las diferencias sexuales en la respuesta al estrés y sus mecanismos en población que ha sufrido violencia en la infancia. Finalmente, cabe mencionar que las alteraciones en la regulación del cortisol también alteran otros sistemas importantes de neurotransmisión, entre ellos, el dopaminérgico, que participa en la regulación de la tendencia a la adicción a drogas de abuso y consumo de alcohol (19-20, 50), por lo que su estudio en poblaciones vulnerables resulta necesario.

Dentro de las limitaciones observadas tenemos que solo se encontró literatura en inglés, ninguna de ellas en países de Latinoamérica, con lo que se evidencia la necesidad de llevar a cabo estos estudios en la región. Es necesario realizar estudios con mayor sistematización en el diseño y reporte de resultados, principalmente los que se refieren a la hiporreactividad, a las diferencias de género y a los grupos de comparación y control, ya que la hiper o la hiporreactividad deberían estar definidas en función a la respuesta de un control de población saludable. Además es necesario estandarizar variables como, el lapso de tiempo que las personas estuvieron expuestas a violencia, la edad de inicio de la exposición, datos que se obtienen de manera retrospectiva. Todo ello facilitaría la realización de metanálisis.

En conclusión los resultados de esta revisión nos permiten sugerir que es posible considerar la hiperreactividad del cortisol como un biomarcador para el diseño de intervenciones y tratamientos farmacológicos de personas que sufren trastorno de estrés postraumático y depresión como consecuencia de sufrir violencia temprana. Dado que la severidad de los trastornos puede influir en la alteración del eje HHA, estudiar la relación entre la RC, el sexo, la severidad y tipo de violencia, puede aportar datos relevantes sobre las afectaciones en población adulta para proponer políticas de salud así como para implementar tratamientos eficaces. Sin embargo, se requieren más estudios, principalmente longitudinales y retrospectivos, para investigar si la respuesta atenuada podría ser un factor de riesgo en la etiología de desórdenes psiquiátricos o si se trata de una capacidad de resiliencia y factor protector.

## Agradecimientos.

Este trabajo fue apoyado por el Programa Nacional “Igualdad entre los Hombres y las Mujeres 2015” y por el Grupo Interdisciplinario de Investigación sobre Violencia, Salud Mental y Género del Instituto Nacional de Psiquiatría “Ramón de la Fuente Muñiz. Ciudad de México. México. Agradecemos al Dr. Rodolfo Rivas y a la Dra. Luciana Ramos por los valiosos comentarios al trabajo, a Claudia Ahumada por la corrección de estilo.

## Declaración.

Las opiniones expresadas en este manuscrito son responsabilidad del autor y no reflejan necesariamente los criterios ni la política de la *RPSP/PAJPH* y/o de la OPS
